# Manipulation of Mononuclear Phagocytes by HIV: Implications for Early Transmission Events

**DOI:** 10.3389/fimmu.2019.02263

**Published:** 2019-09-24

**Authors:** Kirstie Melissa Bertram, Orion Tong, Caroline Royle, Stuart Grant Turville, Najla Nasr, Anthony Lawrence Cunningham, Andrew Nicholas Harman

**Affiliations:** ^1^School of Medical Sciences, University of Sydney, Sydney, NSW, Australia; ^2^Center for Virus Research, The Westmead Institute for Medical Research, Sydney, NSW, Australia; ^3^HIV Biology, Kirby Institute, Kensington, NSW, Australia; ^4^The University of New South Whales, Sydney, NSW, Australia

**Keywords:** HIV, mononuclear phagocyte, dendritc cell, macrophage, transmission, interferon

## Abstract

Mononuclear phagocytes are antigen presenting cells that play a key role in linking the innate and adaptive immune systems. In tissue, these consist of Langerhans cells, dendritic cells and macrophages, all of which express the key HIV entry receptors CD4 and CCR5 making them directly infectible with HIV. Mononuclear phagocytes are the first cells of the immune system to interact with invading pathogens such as HIV. Each cell type expresses a specific repertoire of pathogen binding receptors which triggers pathogen uptake and the release of innate immune cytokines. Langerhans cells and dendritic cells migrate to lymph nodes and present antigens to CD4 T cells, whereas macrophages remain tissue resident. Here we review how HIV-1 manipulates these cells by blocking their ability to produce innate immune cytokines and taking advantage of their antigen presenting cell function in order to gain transport to its primary target cells, CD4 T cells.

## Introduction

Mononuclear phagocytes (MNP) are a group of antigen presenting cells (APC) which include monocytes, Langerhans cells (LC), dendritic cells (DCs), and macrophages. Circulating blood monocytes and DCs are bone marrow derived whereas LCs and tissue resident macrophages, such as Kupffer cells in the liver and those in lungs, kidneys, are seeded during embryogenesis. However, both LCs and macrophages can be replenished by infiltrating monocytes. In tissue, MNPs are the first immune cells to interact with invading pathogens such. HIV is transmitted across the various skin or mucosal tissues that comprise the genital and anorectal tracts which differ in their anatomy. The foreskin, glans penis, labia, vagina, ectocervix, fossa navicularis, and anal canal contain a stratified squamous epithelium mostly consisting of differentiating layers of keratinocytes resulting in a physically impregnable barrier against incoming pathogens. However, HIV transmission across this surface has been shown in human *ex vivo* genital tissue explants from the vagina, ectocervix, and foreskin (1–6). In type I mucosal tissue (urethra, rectum, and endocervix) a single layer of columnar epithelium separates the lamina propria from the lumen and HIV has been found to cross this barrier in mucosal explants and interact with MNPs (7, 8).

The primary role of MNPs is to detect incoming pathogens via an array of pathogen binding receptors expressed on their surface. These include Toll-like receptors (TLR) and lectin receptors such as C-type lectin rectors (CLR) and Sialic acid-binding immunoglobulin-type lectins (Siglec). In response to pathogens binding TLRs, MNPs produce an array of innate immune cytokines including type I and III interferons, chemokines, and proinflammatory cytokines. Type I and III interferons trigger the induction of a wide array of interferon stimulated genes (ISG), both in infected and bystander cells, which encode antiviral proteins to help cells combat the viral infection. Chemokines such CCL3-5, act to recruit other immune cells to the site of infection and proinflammatory cytokines, such as tumor necrosis factor (TNF), interleukin (IL)-1B, and IL-6, act to activate these cells to prepare them to elicit a potent immune response. Thus, many successful invading pathogens, such as HIV, have evolved mechanisms to block the ability of cells to produce innate immune cytokines, or evade them.

Following detection of pathogens via CLRs, MNPs rapidly endocytose (LCs and DC) or phagocytose (macrophages) them, destroying them via acid proteolysis. Macrophages remain in tissue, but LCs and DCs undergo a process of maturation triggering them to migrate to the draining lymph nodes where they present pathogen antigens to CD8 and CD4 T cells via MHC-I and –II, respectively. They then mediate an immune response against pathogens or immune tolerance toward commensal bacteria. As CD4 T cells are the main HIV target cells in which the virus undergoes active replication, LCs and DCs provide HIV with a delivery vehicle from the site of initial exposure to the lymph nodes where the virus has access to a high concentration of CD4 T cells. However, the virus must avoid destruction by acid proteolysis in the process. It is currently unclear if macrophages can perform a similar function within the mucosa at the site of initial exposure.

In this review, we describe how HIV-1 manipulates MNPs in order to facilitate successful transmission. We review how HIV-1 blocks interferon induction in MNPs while at the same time inducing the expression of specific ISG. We also describe how HIV-1 modulates DC maturation such that it triggers migration to draining lymph nodes but avoids destruction by acid proteolysis. Finally, we describe two independent mechanisms by which HIV-1 is transferred from MNPs to CD4 T cells.

## Mononuclear Phagocytes Subsets and Sexual Transmission of HIV

MNPs consist of monocytes, LCs, DCs, and macrophages.

### Monocytes

Monocytes are found in blood only and although there is some evidence that certain monocyte subsets (especially CD16^+^ monocytes) may represent HIV target cells, they are not discussed in this review which is focused on HIV transmission within anogenital tissues where it now occurs in the vast majority of cases.

### Dendritic Cells

Dendritic Cells are potent APCs and differentiate from bone marrow derived precursors. They can be split into three key subsets: plasmacytoid DCs (pDC) and two subsets of myeloid or conventional (c) DCs named cDC1 and cDC2.

### pDCs

The primary role of pDCs is to migrate to inflamed tissue and secrete large quantities of Type 1 interferons to combat viral infections. However, they have also been shown to act as APCs and stimulate CD4 T cells, earning them their descriptor as a type of DC. These cells express the HIV entry receptor CD4 as well as the two key chemokine receptors CXCR4 and CCR5 and HIV can enter these cells via the classical CD4 entry pathway or can be taken up via dynamin endocytosis where the viral RNA is detected by TLR7 (9–11) triggering type I interferon induction. As they are found in inflamed tissues only they are not generally regarded as key players in initial HIV transmission, and will not be discussed in the review in detail which focuses on myeloid cells. However, we refer the reader to a recent review on the involvement of pDCs in infection and pathogenesis (12). However, two points should be noted. Firstly, pDCs have previously been defined as CD11c– CD123^+^ CD303/BDCA2^+^ cells but recently this group of cells has been shown to be made up of a heterologous population of *bona fide* pDCs which secrete Type I interferons, and a small myeloid population that can be distinguished by expression of Axl and Siglec-6, hence named AS DCs (13, 14). It is the AS DC myeloid component that act as APCs, but their potential role in HIV transmission and pathogenesis has yet to be investigated. Secondly, recently it has become clear that sexual transmission of HIV is strongly associated with inflammation (15–17), especially in Sub-Saharan Africa. This is a key concern as some current prevention strategies are not as effective in the context of an inflamed mucosa (18–20). Therefore, the role that pDCs, AS DCs, and other potential inflammatory MNPs should be investigated.

### cDC1

cDC1 are present in the dermis, lamina propria, anogenital skin, and mucosa and were originally defined by their expression of CD141/ BDCA3. However, this marker can also be expressed on cDC2 under some circumstances and cDC1 are now better defined by their unique expression of CADM1, IRF8, Clec9a, and XCR1 (13). cDC1 are able to detect dead and dying cells via their unique CLR Clec9a which binds F-actin (21, 22) and importantly can cross-present antigen to CD8 cells or directly to CD4 T cells. Few studies have investigated these cells in the context of HIV but a recent study showed they express high levels of the retroviral restriction factor SAMHD1 and are resistant to infection (23). They will not be discussed further, however it is of note that poly(I:C) was recently shown to up regulate the expression of costimulatory molecules in cDC1 in response to HIV peptides making them important to consider for potential vaccine design (24).

### cDC2

cDC2 are also present in the dermis and lamina propria and are more abundant than cDC1. They can be defined by their expression of CD1c, IRF4, and high expression of CD11c. In skin only they also express CD1a and a minor population have been shown to express the CLR langerin (25). cDC2 express the HIV-1 entry receptors CD4 and CCR5 as well as a wide range of pathogen binding CLRs and cDC2 cell counts are correlated with a high risk of rapid disease progression during early HIV-1 infection (26). Despite this, until recently, these cells had not been studied in the context of HIV transmission. However, in a recent study Bertram et al. (27), showed that these cells are present at the epithelial surface of human anogenital tissues where they capture HIV and then transfer the virus to CD4 T cells. Furthermore, they showed that these cells predominate over LCs in these tissues and thus could be the primary HIV transmitting MNP. In another study DCs have been reported to exist in vaginal stratified squamous epithelium which the authors refer to as Vaginal Epithelial Dendritic Cells (VEDC) (28). These are almost certainly the cDC2 described by Bertram et al. (27).

### Langerhans Cells

Langerhans Cells are found within the stratified squamous epithelium which forms the outermost layer of anogenital tissues comprised of skin (anal verge, labia, and outer foreskin) or Type II mucosa (vagina, cervix, inner foreskin, glans penis, fossa navicularis, and anal canal). These were originally believed to be a type of DC however more recently some groups have argued that they are more macrophage-like (6, 29–31). In terms of function they act like DCs in that they migrate out of tissue in response to pathogen exposure, and present antigens to CD4 T cells in the lymph node. However, ontologically they are more like macrophages as both cell types have an embryonic origin but can be reseeded after inflammation by two waves (32, 33). The first wave is a transient population that appear to be derived from circulating monocytes. The second wave establish long-term LCs derived from precursors of an unknown origin (34). In regards to this Bertram et al. recently described a human epidermal cell that is phenotypically almost identical to LCs but can be differentiated by their lower expression of CD33. These CD33^low^ LC-like cells differ to LCs in their morphology and are also functionally very weak APCs. Thus, these CD33^low^ cells could represent a LC precursor. DCs on the other hand are exclusively derived from bone marrow derived progenitors (31). Therefore, LCs are best grouped as their own MNP subset. Prior to the very recent studies by Bertram et al. (27) and Pena-Cruz et al. (28), LCs were believed to be the only MNP subset found within the epidermis and as such much research has been conducted on these cells as they have been considered to be the first cells that interact with HIV (6). Recently, an increase in “LCs” (CD1a^+^ expressing cells) in human inner foreskin explants after 24 h virus exposure has been described (35). This was repeated with macaques *in vivo*, however, these cells may be more like the recently described cDC2/VEDCs than *bona fide* LCs as CD1a does not discriminate between LCs and cDC2 (27).

LCs bind HIV via their unique CLR langerin and can be infected via CD4 and CCR5 but their role in transmission is still controversial. Some groups argue they play a critical role in transmission by directly transferring HIV to CD4 T cells (1–3, 36), while another group has argued they form a natural barrier to HIV and degrade the virus in Birbeck granules (37, 38); the latter may be explained by isolation methods using trypsin which cleaves CD4, preventing infection. Importantly in anogenital tissue, langerin^+^ cells have been shown to express CCR5 and CXCR4 in human foreskin. Higher CCR5 expression was found on langerin^+^ cells present in the inner compared to the outer foreskin (39), so these cells are prime target cells for HIV.

### Macrophages

The primary role of macrophages is the phagocytosis and killing of pathogens at the site of infection and in tissue homeostasis. They are relatively weak APCs, unlike DCs, but similar to LCs they are seeded in tissue during embryogenesis but can also be replenished within tissue by infiltrating monocytes. Traditionally they have been defined by their expression of CD14 and the transcription factor FXIIIA and high autofluorescence. Recently, four distinct subsets of macrophages (Mf1, Mf2, Mf3, and Mf4) have been defined in the human small intestine which have distinct phenotype, function, and anatomical location within the mucosa (40). It will be of interest to investigate whether these subsets interact with the virus early post anorectal exposure and if they can become infected.

Macrophages express relatively low levels of CD4 but moderate levels of CCR5 unlike circulating CD14^+^ monocytes which are CCR5^−^ (41). However, once they enter tissue and differentiate into macrophages they upregulate CCR5 and become susceptible to HIV infection. In contrast circulating CD16^+^ monocytes are CCR5^+^ and susceptible to HIV. Macrophages can be infected by cell-free HIV or cell-to-cell transmission from infected CD4 T cells via virologic synapses, or by selective phagocytosis of infected CD4^+^ T cells, leading to efficient infection (42, 43). Macrophages can also bind to HIV via mannose receptor (MR) (44) and DC-SIGN which act as concentrating receptors prior to binding to CD4 and CCR5(45). The levels of productive infection of monocyte derived monocytes (MDMs) by clinical isolates is HIV strain and donor dependent as shown by studies with identical twins (41, 46). Furthermore, as HIV infection progresses to immunodeficiency and AIDS the CCR5 utilizing strains become more infectious for MDMs (47), this being predominantly determined by enhanced HIV envelope binding to CCR5 (see below) (5). Prior to the availability of combination antiretroviral therapy the ability of HIV, SIV, or SHIV to persist with noncytopathic infection in macrophages in brain, lymph nodes, gut, spleen, and marrow was well-described (48–52).

More recently there has been intense interest in macrophages for their potential secondary role as sites of persistent latent infection, after resting memory CD4 T cells, during combination ARV therapy (cART) (53). Recent (and older) studies in SIV-macaque models and in humans have shown persistent infectious virus in alveolar macrophages (43, 48), lymph nodes (54), spleen and liver before cART, and persistent viral DNA during ART, but infectious virus was not observed in macaque and human alveolar macrophages (43) or human liver (55) while under or after treatment with cART. In the brain, infectious HIV persists mainly in microglial cells and perivascular macrophages. Noninfectious HIV DNA is in astrocytes and is inversely proportional to peripheral CD4 T cell counts. The effect of cART, which often penetrates poorly into brain, seems to incompletely suppress HIV infectivity (56). Severe HIV-associated neurocognitive disorders (HAND) has become less common in the cART era but milder forms of HAND persist. Whether this represents inadequate cART suppression of HIV in brain myeloid cells, or whether there is a modified form of latency, requires further studies. In blood CD16^+^ monocytes express higher levels of CCR5 than CD14^+^ CD16^−^ monocytes. Their transport of virus into tissues and differentiation into macrophages bearing HIV, especially in to CD163 perivascular macrophages also needs further definition (57).

### Are Non-autofluorescent CD14^+^ Cells Macrophages or DCs?

It was previously believed that non-autofluorescent CD14 expressing tissue cells were DCs. However, in skin, these cells have recently been shown to be more transcriptionally similar to macrophages and have been redefined as monocyte-derived macrophages which are derived from CD14^+^ monocytes that migrate into tissue from the blood (58). Thus, in skin, all CD14 expressing cells are now defined as macrophages. Both macrophage populations express Dendritic Cell-Specific Intercellular adhesion molecule-3-Grabbing Non-integrin (DC-SIGN)/CD209^+^. They are concentrated in lymphoid aggregates more commonly found in inner than outer foreskin (39).

Whether or not intestinal non-autofluorescent CD14 expressing cells are also macrophages and not DCs is currently a very interesting topic in the field of HIV as there are a plethora of studies using intestinal tissue showing that these cells are key target cells for HIV via their CLR, DC-SIGN. These cells have been shown to extend dendritic process through the columnar epithelium to sample pathogens in the intestinal lumen (59, 60). They have been specifically shown to sample HIV in this fashion and then transfer the virus to CD4 T cells in the underlying lamina propria (4, 61). Similarly, these cells have been shown to be key HIV target cells in the rectum (62). Finally CD11c^+^CD14^+^ cells they have recently been shown to capture HIV in the female reproductive tract (63). Although they may be transcriptionally similar to macrophages, functionally they may be more similar to DCs and, like LCs, these may need to be redefined as a new type of MNP.

## Monocyte Derived Dendritic Cells and Macrophages

As MNPs are so difficult to isolate from tissue and also undergo maturation as a result of their extraction (64), by far the majority of studies have been carried out on *in vitro* derived monocyte-derived DCs (MDDC) and MDM. These cells can be generated in larger numbers in an immature state by culturing blood CD14^+^ monocytes in IL-4 and GM-CSF for MDDCs, or in MCSF or human serum only for MDMs. Interestingly MDDCs were shown to be most transcriptionally similar to tissue CD14^+^ MDMs (then referred to as CD14^+^ DCs), which correlates with their mutual expression of DC-SIGN (65). By far the majority of studies described below have been conducted in these model MNPs.

## DC Transfer of HIV to CD4 T cells

DCs have been shown to transfer HIV to T cells in two ways. First-phase transfer (also termed trans-infection) involves binding of a PRR on the MNP, holding the virus in a nondegradative compartment, then delivering it to a T cell once a virological synapse is formed. Second-phase transfer (also termed cis-infection) involves infection of the MNP via CD4 and CCR5 co-receptor CCR5 and *de novo* synthesis of the virus before transferring the virus to a T cell. Second-phase transfer can be blocked by pre-incubating the cells with inhibitors to entry or reverse-transcriptase (RT) (e.g., the CCR5-binding inhibitor Maraviroc, or the RT inhibitor AZT), whereas first phase cannot (36, 66).

First-phase transfer was initially attributed to the CLR DC-SIGN which has been shown to bind gp120 (67). After uptake some DC-associated HIV-1 evades the endocytic degradation pathway by trafficking to a tetraspanin (CD81^−^) enriched protective environment from where infectious particles are specifically released to T cells upon DC: T cell contact (68). DC-SIGN, although present on MDDCs, has been shown in tissue to be expressed predominantly on macrophages (58). Other CLRs have now been also shown to bind HIV and implicated in first-phase transfer including: langerin (CD207) (36) expressed at high levels on Langerhans cells (LCs) and at lower levels on a subset of cDC2s (25), mannose receptor (MR or CD206), and DC immunoreceptor (DCIR or CLEC4A) (69).

Siglec-1 (or CD169) (70) reviewed in Akiyama et al. (71) and expressed on cells of myeloid lineage has been shown to take-up HIV into a storage compartment and enhance first-phase transfer by MDDCs, MDMs, and tissue-derived (tonsil) cDC2s (70). Siglec-1 has been shown to work in conjunction with tetherin to form these virus-containing compartments (72).

Tetherin (BST-2 or CD317) is an IFN-I inducible restriction factor that blocks the release of enveloped viral particles from infected cells. Tetherin has been shown to restrict the transfer of HIV-1 from myeloid cells to CD4^+^ T cells (73–75). Tetherin is antagonized by SIV Nef in non-human primates (76–78). Humans, however, have a deletion in tetherin that prevents the SIV Nef antagonism. Interestingly, different HIV-1 strains have evolved to antagonize tetherin by utilizing different accessory proteins and counteracting tetherin to different degrees. The HIV-1 group M strains (responsible for the global AIDS epidemic) have gained the ability to utilize Vpu to antagonize tetherin (77, 79, 80). HIV-1 group O Nefs target a region of tetherin that leads to down modulation of tetherin but this had no effect on MDM:T cell transfer (81). Furthermore, HIV-1 group O strains have been identified that use both Nef and Vpu to target tetherin (82). Tetherin antagonism appears to play a major role in transmitted/founder strain resistance to type I IFN (83). A Vpu mutant virus that abrogated tetherin antagonism induced comparable gene expression to wild-type, whereas a Vpu *null* mutant induced very different gene expression in CD4^+^ T cells (84).

Lectins can also be involved in second-phase transfer, which is dependent on productive infection of the MNP. Hijazi et al. found that DC-SIGN can increase the stability of the gp140: CD4 complex, enhancing infection (45) whereas langerin does not. DCIR (69) has been shown to enhance second-phase transfer and in MDMs Siglec-7 has been shown to enhance infection (85). Interestingly, MR expression, has been shown to be inhibited by HIV-1 in macrophages [as well as by HIV-2 and SIV reviewed in (86)]. Without downregulation of MR, HIV-1 virions were held, stuck on the surface of MDMs and downregulating MR allowed more virus to be released from the surface, aiding spread (87).

As productive infection is needed for second phase transfer of HIV, it is pertinent to examine what factors influence HIV productive infection of MNPs. It has long been identified that HIV-1 strains display tropism to different cell types, with some able to infect T cells but not myeloid cell types (macrophages having been the most studied). Strains that are able to infect myeloid cells or macrophages have been termed M-tropic. Earlier studies found macrophage-tropic viruses are able to utilize CCR5 and low levels of CD4 (88–90). This is possibly due to gp120 polymorphisms that increase macrophage tropism through enhanced interactions with CCR5 (91). Although this broadening of tropism my come at the cost of lower infectivity (92). Adding to the complexity, a recent study has highlighted that CCR5 exists in heterogeneous conformations and oligomerization states. Different subpopulations of CCR5 were preferred by divergent viruses (93) and importantly, macrophages and T cells expressed different subpopulations of CCR5 and it is likely another factor driving virus strains to be macrophage-tropic or T cell-tropic. It remains to be studied what subpopulations of CCR5 are expressed by all the various tissue myeloid cells that exist at the site of infection or if indeed this is the main determinant of M-tropism.

## Manipulation of Maturation, Migration, and Lysosome Function by HIV

After detecting pathogens via TLRs and CLR's DC undergo a process of maturation whereby their function changes from cells specialized in antigen capture to those specialized in antigen presentation. Thus, pathogen binding receptor expression is down-regulated leading to a reduced ability for anigen detection and endocytic uptake. Secondly, CCR7 is upregulated which allows for chemotactic migration to the lymph nodes along CCL19 and CCL21 gradients. Thirdly, various surface molecules are up-regulated that facilitate DCs–T cell interactions such as those involved in antigen presentation (MHC-II), T cell activation (CD40, CD80, CD83, CD86), and cell adhesion (CD54/ICAM-1). This allows for the formation of clusters of mature DCs and T cells within the lymph nodes. Such clusters require the formation of an immunological synapse (94, 95) which requires actin cytoskeleton remodeling of the and an interaction between LFA1 on the T cell and CD54 on the DC. Within the synapse costimulatory and antigen presentation molecules on the DC membrane, and their cognate receptors on the T cell membrane, become concentrated (94, 95), and this contact region facilitates T cell activation and antigen presentation. If the DCs are HIV infected with HIV then a virological synapse is formed with T cells instead, which allows for transfer of HIV from the DC to the T cells (96, 97). Though similar, the immunological and virological synapse (94, 95) differ in their complement of adhesion molecules and other proteins (98).

As DCs migrate from the site of pathogen exposure to the lymph nodes which are rich in the primary HIV target cells, CD4 T cells, this makes them an ideal delivery vehicle for HIV to exploit. Indeed, it is well-known that DCs enhance infection of T cells so efficiently that they become insensitive to the anti-viral drugs tenofovir and raltegravir (99). Mature DCs form DC:T cell conjugates more readily than immature DCs (96). However, there has been controversy in the literature as to whether HIV can trigger DC maturation, some arguing in favor (100–102) and some against (103–105). To address this controversy Harman et al. (102) carried out a detailed and through study and showed that HIV induces both MDDCs and *ex vivo* LCs (derived using collagenase) to undergo a process of partial maturation via two independent mechanism resulting in an increased migratory and T cell stimulatory capacity. As a key differential between the conflicting studies was the way in which HIV virus stocks were produced, they then went on to investigate this process more fully. By comparing raw virus stocks to highly purified ones a follow up study showed that one of the mechanisms of HIV induced maturation was driven by microvesicles in the viral inoculum and the other was dependent on productive infection of the DCs. They also showed that HIV drive increased CD54 (ICAM-1) expression which lead to increased DC: T cell interactions (106). Other studies have shown that cross-talk between infected MDDCs and T cells decreases the restriction factor SAMHD1 in the MDDCs, promoting efficient viral replication (107). This was dependent on viral synapses as blocking CD54 reduced the effect.

A key aspect of DC maturation is the destruction of the pathogen by acid proteolysis in lysosomes and subsequent loading of antigen fragments on MHC-II. This process is mediated by the cathepsin and cystatin families of proteins. Using microarrays Harman et al. were able to show in MDDCs that HIV specifically down-regulates the expression of specific cathepsin proteins that degrade pathogens and up-regulate specific cystatin proteins that act to negatively regulate this process. Furthermore, using enzyme function assays they showed that HIV was able to disrupt the enzymatic activity of cathepsins to inhibit lysosomal degradation and thus protect the virus from degradation by at least two independent mechanisms. These two effects of HIV on DC biology are part of a wider spectrum, shown by the fact that HIV-1 induces two waves of gene expression in MDDCs that correspond to the two phases of HIV transfer to T cells (108).

HIV-1 has also evolved mechanisms to avoid complement-mediated destruction by incorporating regulators of complement activation during the budding process and by binding the fluid-phase factor H (109, 110). Complement-opsonized HIV-1 triggered T592 phosphorylation of SAMHD1, allowing increased HIV production and initiating the strong maturation and co-stimulatory capacity of DCs (111).

## Manipulation of the Interferon System in Mononuclear Phagocytes by HIV

IFNs represent a family of critical effector cytokines that underpin both innate and adaptive antiviral immunity, and have been grouped into three distinct classes (112, 113). Type I IFNs (IFN-I) encompass IFN-α (which has thirteen human sub-types), IFN-β, IFN-ε, IFN-κ, and IFN-ω, which all signal through the IFNA receptor (114, 115). IFN-ε is constitutively expressed in the female reproductive tract (116) but it has also been shown to be produced in response to viral infection in both MDDCs and MDMs (117). Type II IFN consists of a single cytokine, IFN-γ, which is mainly secreted by T cells, NK cells and innate lymphoid cells. Type III IFNs consists of IFN-λ1-4 (118) and induce IFN-I-like antiviral activity as well as sharing features with the IL-10 family (113, 119, 120). Whilst IFN-I have a broad expression and activity range due to ubiquitous expression of the IFNA receptor, the expression of the IFN-λ receptor is generally limited to epithelial cell surfaces (121).

Type I and III IFNs can act through autocrine and paracrine mechanisms to induce the expression of numerous interferon-stimulated genes (ISGs) in both host and neighboring cells, collectively enacting an antiviral state. The induction of these ISGs can lead to degradation of viral RNA/DNA and the inhibition of viral gene expression, protein synthesis, virion assembly and release, with marked variation across cell types (122–124). In addition, IFN-I can enhance immune cell activation and effector functions, which includes promoting an antigen-presenting phenotype in DCs and macrophages (125, 126), stimulating CD8^+^ T cell clonal expansion and NK cell activation (127–132) as well as B cell class switching and affinity maturation (133).

## Pathways of IFN-I production in Mononuclear Phagocytes

**Figure 1:**
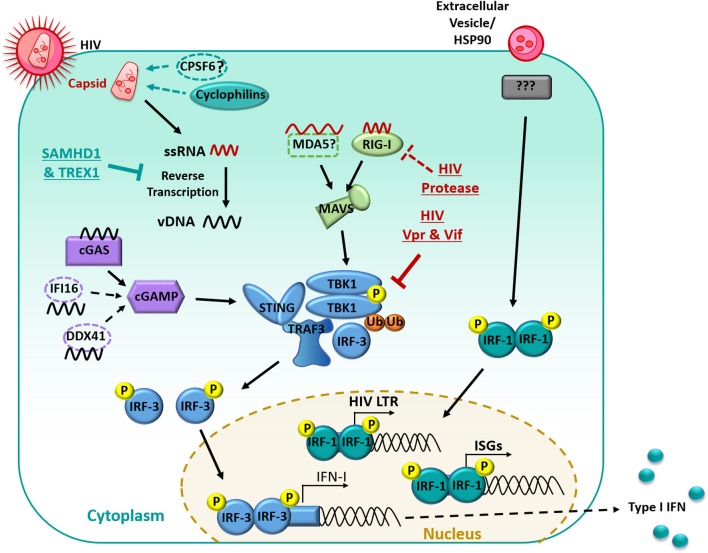
Manipulation of the interferon signaling pathway in myeloid cells. HIV-1 infection is restricted by the host proteins SAMHD1 and TREX1, limiting Reverse transcription. RNA is detected primarily by RIG-I and signaled through MAVS. Reverse Transcribed viral DNA is primarily detected by cGAS and signals through cGAMP. PRR recognition signals are then transduced through STING which results in the downstream clustering of TBK1 which complexes with TRAF3 and IRF-3. TBK1 then undergoes ubiquitination and auto-phosphorylation and can then phosphorylate IRF-3. Phosphorylated IRF-3 then dimerises and translocates to the nucleus, where it induces the expression of IFN-I. HIV-1 limits the IFN response in many ways, including undergoing reverse transcription with in the capsid, the capsid also recruits host proteins (primarily cyclophilins) to shield HIV-1 from PRRs. The HIV-1 protease targets RIG-I for degradation. HIV-1 Vpr and Vif prevent the auto-phosphorylation of TBK1 and subsequent translocation of IRF3 to the nucleus. Extracellular vesicles in the HIV-1 inoculum can induce IRF-1 translocation to the nucleus. IRF-1 induces a subset of ISGs and an IRF-1 binding site is also located on the HIV-1 promoter which may enhance subsequent virus production.

During viral infection, IFN-I production is usually triggered in MNPs through the recognition of viral nucleic acids by cytoplasmic sensors of RNA: retinoic acid-inducible gene 1 (RIG-I) and melanoma differentiation-associated gene 5 (MDA5), or DNA: mainly cyclic GMP-AMP synthase (cGAS), with some possible contribution from interferon-γ-inducible protein 16 (IFI16) and polyglutamine binding protein 1 (PQBP1) (134–136) (summarized in Figure 1). Whilst RIG-I and MDA5 signal through the adaptor mitochondrial antiviral-signaling protein (MAVS) upon recognition and binding of viral RNA, activation of cGAS, IFI16, or PQBP1 by viral DNA instead leads to signaling through the adaptor stimulator of IFN genes protein (STING). Both MAVS and STING activation result in the downstream clustering of TANK binding kinase-1 (TBK1), which forms a complex with TNF receptor-associated factor (TRAF3) and interferon-regulatory factor (IRF)-3 (137). TBK1 subsequently undergoes ubiquitination and trans-autophosphorylation, by which it becomes self-phosphorylated, and can then phosphorylate IRF-3 (138). Following phosphorylation, IRF-3 dimerises and translocates to the nucleus where it induces the expression of IFN-β by binding to IFN-stimulated response elements in the promoter of various genes, including *IFNB* and other ISGs. This then leads to autocrine and paracrine signaling through the IFNA receptor which triggers IRF7 transcription, mediating a positive feedback loop of IFN-I production (139). Along with IFN-β, multiple subtypes of IFN-α are produced during this second wave of gene transcription (140).

The importance of IFN-I on the course of HIV infection has been well-established but presents an apparent paradox on disease outcome, namely an apparent protective function during the earliest stages of infection, but contribution to pathogenesis during and after the transition to chronic infection. Studies performed in rhesus macaques and humanized mice (141–143) examining early timepoints after infection (up to 12 weeks post infection) have highlighted the importance of an early IFN-I response to SIV/HIV, with the inhibition of IFN-I signaling resulting in increased reservoir size and accelerated progression to end-stage disease. Indeed, exogenous addition of IFN-I prior to SIV infection in rhesus macaques delays SIV acquisition and necessitates an increased number of challenges for transmission to occur (142, 144). Furthermore, studies looking at transmitted HIV isolates have reported that founder viruses that establish infection are largely more resistance to the inhibitory effects of IFN-I and ISGs on viral replication compared to their non-transmitted counterparts isolated from the same individual (145, 146). This relative IFN resistance suggests that early IFN-I constitutes a major selective barrier against the initial virus the host is exposed to and helps to protect against infection during the acute phase. In complete contrast, protracted production of IFN-I into chronic HIV infection exacerbates disease progression (147, 148). This has been observed in non-pathogenic SIV infection of natural hosts such as African Green monkeys and Sooty Mangabeys where a strong IFN-I signature occurs early but then dissipates (148–150). On the other hand, pathogenic infection in rhesus macaques shows sustained IFN-I production, including through to chronic infection. A similar parallel can be observed with HIV-1 and the less pathogenic HIV-2, where HIV-2 induces a shorter burst of IFN-I production *in vitro* (151). Continued administration of IFN-I in rhesus macaques during SIV infection also leads to desensitized IFN signaling and increased CD4^+^ T cell viral load and depletion, consistent with the immunopathology of chronic immune activation (142). These studies noting the disease association with excess long-term IFN-I are also well reflected in human individuals infected with HIV, where elevated plasma levels of IFN-α has been demonstrated to correlate with plasma viral load, hyperimmune activation and progression to AIDS (152–154).

## Evasion and Inhibition of the IFN-I Response in Mononuclear Phagocytes by HIV

Aside from its genomic RNA, HIV also generates proviral cDNA and post-integration mRNA as part of its retroviral lifecycle, which can all potentially stimulate host nucleic acid sensors to initiate IFN-I production. Given the importance of the early IFN response to viral control, HIV has unsurprisingly adopted various strategies aimed at evading immune recognition and inhibiting the induction of IFN-I in its target cells, including in myeloid DCs and macrophages. It is important to note that many studies exploring these inhibitory mechanisms have performed experiments in cell-lines that could not recapitulated across different cell-lines or in primary cells (155–159), or have relied upon the introduction of Vpx, the accessory protein encoded by HIV-2 and SIV that targets SAMHD1 (discussed in the next section) to infect DCs (155, 160). Given the complexity and functional redundancy within the IFN-I signaling pathway, whether these experiments accurately capture the interactions between HIV and MNPs is subject to debate.

### Limited Early Detection of HIV

Firstly, there is a limited ability for host nucleic acid sensors to detect HIV RNA from the incoming virion or cDNA following reverse transcription. HIV undergoes reverse transcription within the capsid following its fusion-mediated entry into the cytoplasmic space, thus shielding the proviral cDNA from detection by host DNA sensors, and limiting host sensing of the viral RNA genome (161). In addition, the capsid only uncoats close to or directly at the nuclear pore, restricting the exposure of viral nucleic acids to the cytoplasmic sensors (161). Work performed in MDMs and the monocyte THP-1 cell line has also suggested that the capsid recruits host proteins (cyclophilins and CPSF6) that further shield HIV cDNA from cGAS sensing (155, 162), although CPSF6 has not been confirmed in follow-up studies. It has also been shown that only RIG-I but not MDA5 can detect HIV RNA leading to reduced downstream signaling through MAVS (163), with a causal mechanism still to be elucidated.

Furthermore, various host restriction factors such as SAM domain and HD-containing protein 1 (SAMHD1) and three-prime repair exonuclease (TREX1) act to inhibit the genomic lifecycle of HIV, but also inadvertently limit the accumulation of proviral cDNA for sensing by cGAS and IFI16. SAMHD1 depletes the pool of intracellular dNTPs thereby minimizing the reverse transcription process, and also potentially degrades incoming HIV RNA via its ribonuclease activity (164, 165). Likewise, TREX1 degrades cytoplasmic and nuclear DNA (in macrophages) and so limits the copies of HIV cDNA for sensing by cGAS and downstream TBK1 signaling (166). Knockdown of TREX1 in a humanized mouse model enabled the restoration of IFN signaling and delayed the acquisition of HIV (167). The importance of these mechanisms that HIV employs to minimize its detection is highlighted in a recent study by Johnson et al. (160) which demonstrated that inhibition of SAMHD1 with Vpx during HIV infection in MDDCs was sufficient to induce expression of a small number of IRF-3-dependent genes, through an increased buildup of intracellular HIV cDNA which in turn allowed for low levels of cGAS stimulation and STING signaling. However, robust stimulation of the cGAS-STING pathway was only achieved at later timepoints or with additional signaling from another inflammatory pathway.

### Inhibition of IFN S*ignaling* by HIV

HIV has also encoded multiple inhibitory mechanisms aimed at disrupting the key sensors and signaling proteins involved in the host IFN response. Multiple studies performed in our lab in MDDCs and MDMs have identified that HIV completely inhibits the induction of all type I and type III IFNs (IFN-α, -β, -ε, -κ, -ω, -λ) up to 96 h post infection, with a corresponding lack of nuclear translocation of IRF-3 (117, 124, 168). Harman et al. (168) demonstrated this could not be solely attributed to aberrant sensing by upstream pattern recognition receptors, given that TBK1 complexation with TRAF3 and IRF3 could still be observed. Instead, the HIV accessory proteins Vpr and Vif physically prevented the autophosphorylation of TBK1 thereby blocking its ability to phosphorylate and activate IRF-3 (168). Infection with Vpr- or Vif-deficient mutants restored the production of IFN-β in MDDCs and MDMs, suggesting that the targeting of TBK1 represents the major inhibitory mechanism of IFN induction by HIV. This phenomenon appears to be unique to myeloid cells, as IRF-3 is directly targeted for cellular degradation in CD4^+^ T cells (169, 170), which does not appear to occur in MDDCs or MDMs (168, 171). As such, strategies that aim to restore IFN signaling by targeting Vpr/Vif or TBK1 may be promising avenues for the design of novel therapeutics.

In addition to the manipulation of TBK1, additional work using MDMs has demonstrated that the HIV protease appears to target RIG-I for lysosomal degradation (163), and that the adaptor protein IPS-1 functioning downstream of RIG-I is transcriptionally down-regulated by HIV (172), but both of these observations require further validation in other MNPs. Further work has also suggested the binding of HIV to DC-SIGN on the cell surface of MNPs can lead to the recruitment of mitotic kinase PLK1, which blocks downstream DEAD box RNA helicase (DDX3)-MAVS signaling (173), but recent reports confirming the importance of other lectin receptors in HIV binding, particularly Siglec1 (71, 72, 174, 175), have cast doubts on the *in vivo* relevance of this process.

In contrast to the global inhibition of type I IFNs in other MNPs (myeloid DCs and macrophages) by HIV, plasmacytoid DCs can secrete IFN-α/β in the *in vitro* presence of HIV and HIV-infected cells (9, 176, 177). pDCs express constitutively high levels of IRF-7 and sense HIV through TLR7 (9), which directly stimulates IRF-7 and type I IFN production and bypasses the requirement for TBK1/IRF-3 activation. It is worth noting that during the early stages of HIV infection in the anogenital mucosa, pDCs are recruited from circulation and have been detected in the cervical mucosa underlying the epithelium in SIV-infected rhesus macaques within 3–4 days (178, 179). Consequently, they likely represent one of the major sources of IFN during acute and chronic SIV and HIV infection (141, 178).

### ISG Induction by HIV

Despite circumventing the production of IFN-I in MNPs, HIV infection still induces the expression of a distinct subset of ISGs in MDDCs and MDMs, in a biphasic manner (160, 171, 180). In the initial phase (between 0 and 24 h post infection), ~25 ISGs are transiently induced by extracellular HIV vesicles in a cell-type specific manner, with the most up-regulated ISGs in MDMs being *Viperin* (also known as *RSAD2*), *IFIT1, IFIT2*, and *IFIT3* (124, 171), and *ISG15, IFIT1*, and *IFIT2* in MDDCs (160). The induction of these ISGs occurred independent of viral replication and could be mainly attributed to extracellular HIV vesicles present in incoming viral inoculum (171), which can also be found *in vivo* in the circulation of HIV-infected individuals (181, 182). This phase of ISG induction by vesicles appears to rely upon IRF-1 and was also independent of IFN-I, being unaffected by knockdown of adaptor proteins involved in IFN signaling (MyD88, TRIF, MAVS, and STING) downstream of toll-like receptor and cytosolic sensors (171). The preliminary round of ISG expression can be induced in MDMs by stimulation with “shed vesicles” from activated T cells (whether HIV infected or not), or the heat shock protein (HSP)90α, present in extracellular vesicles in HIV inoculum (106). The second-phase of ISG induction occurs around 72 h post infection and results from the sensing of newly transcribed HIV mRNA following Tat-driven initiation and elongation of proviral transcription in productively infected cells (160, 171). These mRNA transcripts are then detected by RIG-I, following by downstream MAVS signaling and IRF-1/IR7-mediated ISG induction, leading to much stronger and wider variety of ISGs being expressed. This model of ISG expression is consistent with previous studies that have observed a first phase of ISG production occurring pre-integration and a second, larger phase following integration (106, 108, 117, 136, 160, 168, 171, 180, 183).

Interestingly, the HIV promoter region contains similar IRF-1/IRF-7 binding sites to the ISGs induced in MDMs (168), which leads to speculation that up-regulation of IRF-1 and IRF-7 by HIV results in composite HIV transcription to produce *de novo* progeny but also ISG induction that limits explosive viral replication. This may potentially allow HIV to achieve a low-level productive and non-cytopathic infection of MDMs that enables the virus to exploit these cells as a long-term viral reservoir. In addition, in their study of ISGs induced by HIV in MDDCs, Johnson et al. (160) also show that other inflammatory stimuli such as TLR stimulation combined with HIV infection can augment the IFN response. Whether these patterns of ISGs are altered, or indeed if IFN-I induction is restored during inflammation *in vivo* is important to resolve, given that genital inflammation is associated with enhanced HIV acquisition.

## Concluding Remarks

The combined role of MNPs as both immune sentinels and APCs makes them the perfect vehicle for HIV to achieve transport from the site of infection in the anogenital epithelium to its primary target cells (CD4 T cells) in the submucosa or lymph nodes. In order to do this HIV must manipulate MNP biology in several important ways. Firstly, it shuts down the interferon response which blocks the ability of others cells in the surrounding area to enter an antiviral state. At the same time it induces the expression of specific ISGs contributing to a low persistent non-cytopathic infection. This allows the cells to deliver newly formed HIV virions to CD4 T cells after migration to the lymph nodes which occurs via at least two separate sequential mechanisms. HIV is also able to manipulate the DC maturation process such that it induces migration to the lymph nodes by upregulating CCR7 and enhances DC-T cell interactions (especially via ICAM-1) while at the same time avoiding destruction by disrupting the lysosomal degradation process.

In order to improve preventative strategies such as PrEP and develop an effective vaccine we need a better understanding of how the HIV penetrates and interacts with its target cells in both inflamed and uninflamed anogenital mucosa. As DCs are the key cells of the immune system that present antigens to T and cells to drive the respective adaptive immune responses they are key initial target cells for an HIV vaccine. A critical piece of information will be to determine which specific subsets of MNPs take up the virus and deliver it to which T or B cells. Once this has been determined specific adjuvants could be used for the targeted activation of the correct specific MNP subsets in mucosa (for mucosal vaccines) or draining lymph nodes (in systemic vaccines) to drive the required response.

Defining the interactions between HIV and its mucosal MNP target cells should also assist in developing PrEP regimes which work in inflamed mucosa. Thus, defining the inflammatory HIV target cells and their HIV binding receptor expression profiles, will aid in the development of blocking agents that can be used to block MNP infection in an inflamed mucosa.

## Author Contributions

KB and OT wrote the main body of the review with input from CR, ST, and NN. AH and AC guided design and construction of review.

### Conflict of Interest Statement

The authors declare that the research was conducted in the absence of any commercial or financial relationships that could be construed as a potential conflict of interest.
